# An unintentional complication during an intentional procedure—Sinus of Valsalva laceration during BASILICA

**DOI:** 10.1007/s00392-020-01745-3

**Published:** 2020-09-21

**Authors:** Norman Mangner, Mohamed Abdel-Wahab, Krunoslav Sveric, Utz Kappert, Julia Fischer, Stephan Haussig, Axel Linke

**Affiliations:** 1 Department of Internal Medicine and Cardiology, Herzzentrum Dresden, Technische Universität Dresden, Fetscherstr. 76, 01307 Dresden, Germany; 2 Department of Structural Heart Disease/Cardiology, Heart Center Leipzig at University of Leipzig, Leipzig, Germany; 3 Department of Cardiac Surgery, Herzzentrum Dresden, Technische Universität Dresden, Dresden, Germany; 4 Dresden Cardiovascular Research Institute and Core Laboratories GmbH, Dresden, Germany

Sirs:

Bioprosthetic aortic scallop intentional laceration to prevent iatrogenic coronary artery obstruction (BASILICA) is a promising technique to prevent ostial coronary occlusion during transcatheter aortic valve replacement (TAVR) [1]. BASILICA prohibits coronary artery obstruction by lacerating the leaflet in front of a threatened coronary artery ostium. The conception is that a sliced leaflet spreads after TAVR and creates a triangular space that allows blood flow towards the sinus and coronary artery, which otherwise would have been occluded. The probable advantage of BASILICA over coronary stent protection is that it directly addresses the valve leaflet itself whereas the latter one may be associated with external stent compression, deformation, and thrombosis with only challenging or impossible options of percutaneous reintervention. BASILICA may result in an easier coronary access after TAVR and does not leave additional material in the aortic root [2].

An 82-year-old female patient suffered from severe symptomatic bioprosthetic aortic valve stenosis (Sorin Mitroflow 21 mm) that was implanted 6 years ago. She had a patent left internal mammary graft to the left anterior descending artery, though the circumflex artery (Cx) was unprotected in a left dominant coronary artery system with only a hypoplastic right coronary artery. The HeartTeam decided for valve-in-valve TAVR due to increased surgical risk and porcelain aorta (STS 6.4%). However, she was at risk for coronary obstruction of the unprotected Cx due to the findings in the pre-interventional computed tomography. The combination of a low left main stem take-off (3.8 mm), a low sinotubular junction height (16.1 mm), a distance between the virtual transcatheter heart valve and the coronary ostium of 4.1 mm, and a distance between the virtual transcatheter heart valve and the sinotubular junction of 2.6 mm defined a type IIIB aortic root according to the VIVID classification [3] (Fig. 1a–d). This condition is associated with an increased risk of coronary obstruction following valve-in-valve TAVR, in particular in bioprostheses with externally mounted leaflets as in our case.

**Fig. 1:**
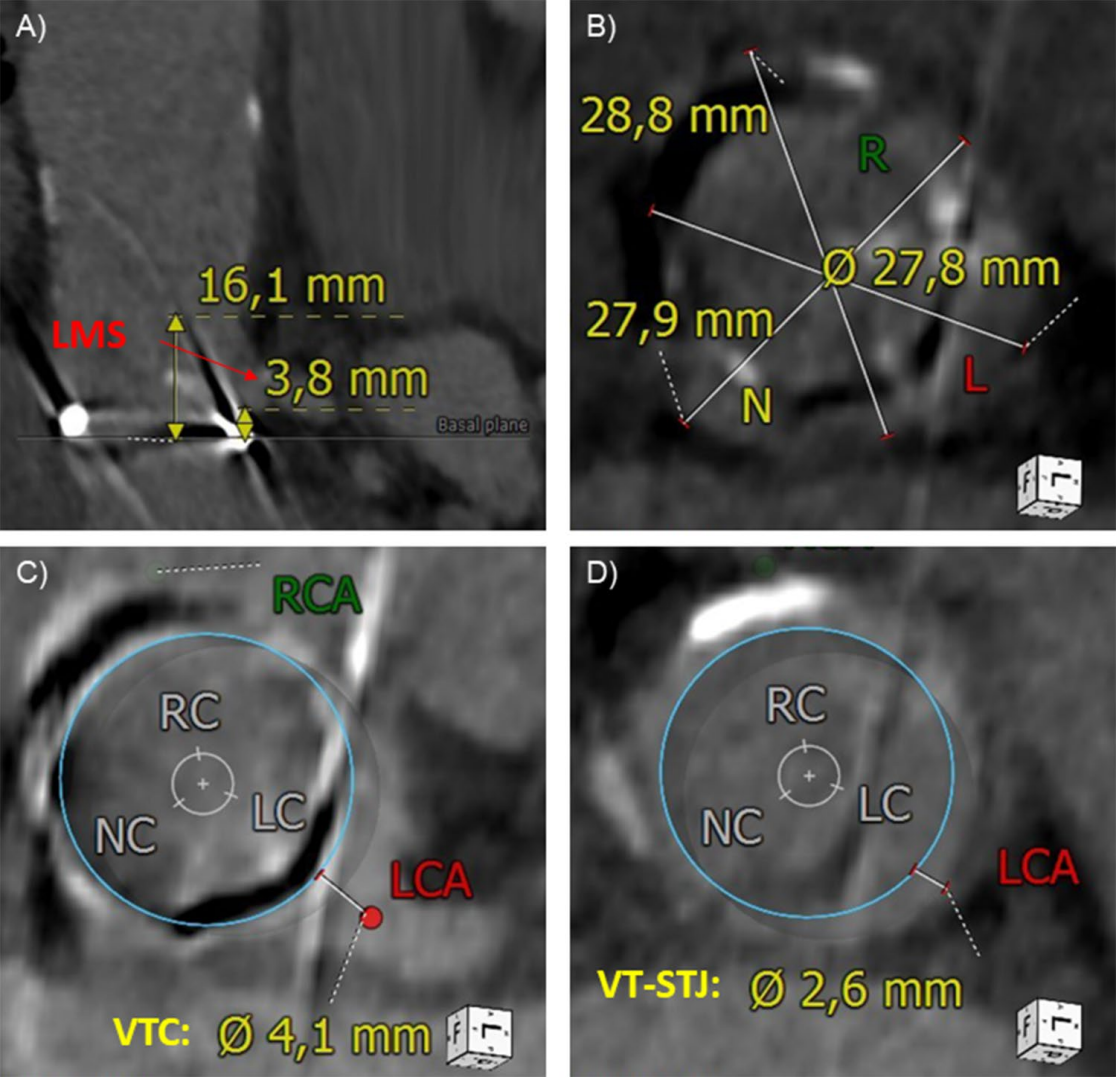
Computed tomography. Low LMS take-off, low STJ height (**a**) and narrow SOV (**b**). Relationship between a virtual THV and the LMS (**c**) and STJ (**d**). *LMS* left main stem, *LCA* left coronary artery, *RCA* right coronary artery, *STJ* sinotubular junction, *THV* transcatheter heart valve, *VTC* distance between the virtual THV and the coronary ostium, *VT-STJ* distance between the virtual THV and the sinotubular junction

A BASILICA procedure was performed under cerebral embolic protection (SENTINEL™, Boston Scientific Corporation, Marlborough, MA). Several traversal attempts with an electrified Astato XS 20 300-cm wire (ASAHI INTECC, Aichi, Japan) were unsuccessful due to severe calcification of the left coronary cusp. The combination of a suboptimal traversal angle with deviation of the wire into the left Sinus of Valsalva (SOV) and prolonged energy application caused laceration of the SOV (Fig. 2a–b). A hematoma developed around the left main stem (LMS) and the posterolateral left atrial wall without pericardial effusion, the patient remained hemodynamically stable (Fig. 2c, ESM Video 1). Further traversal attempts using a different guiding catheter with modified traversal angles were carried out being finally successful (Fig. 3a). ECG changes and concerns of external LMS compression led to angiography prior to laceration revealing embolized material in the Cx, which was treated by drug-eluting stent (DES) implantation (Fig. 3a, b, ESM Video 2). Another DES was positioned in the LMS/Cx followed by laceration of the left coronary cusp and implantation of an EvolutR 23 mm (Medtronic, Dublin, Ireland) (Fig. 3c). Finally, the pre-positioned stent was implanted in anticipation of possible hematoma progression leading to both unimpaired coronary flow and coverage of the laceration, thus stopping the bleeding into the hematoma (Fig. 3d–f, ESM Video 3). Sixteen hours later, the patient developed a localized pericardial tamponade requiring emergency pericardiocentesis (Fig. 4a–c). A prolonged intensive care stay due to pneumonia characterized the further clinical course; however, 34 days after the procedure, she was transferred to a rehab unit.

**Fig. 2:**
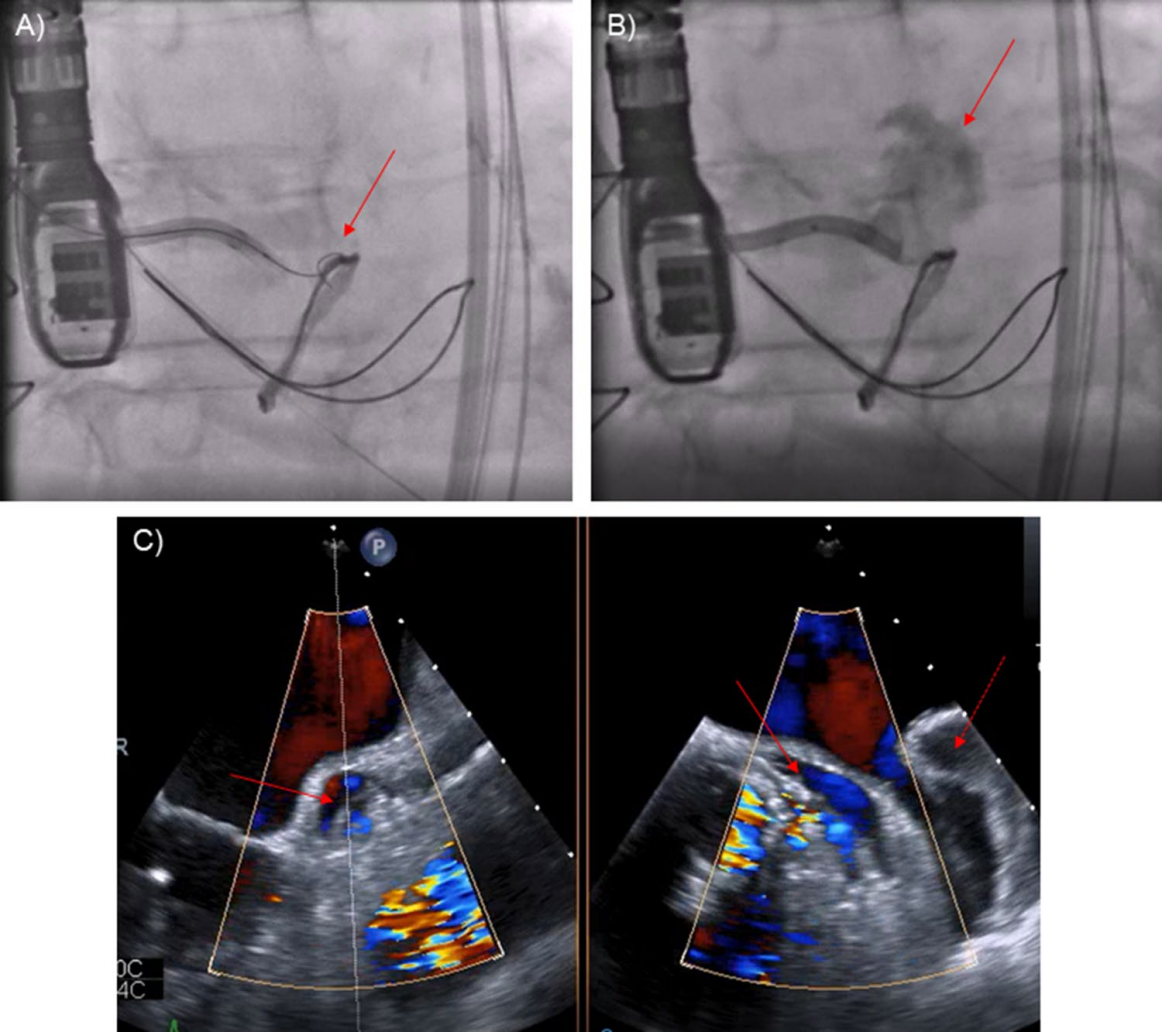
BASILICA procedure I. Traversal attempt with deviation of the electrified wire into the Sinus of Valsalva (**a**, solid arrow) causing laceration confirmed by angiography (**b**, solid arrow) and echocardiography (**c**, solid arrows). Left atrial wall hematoma (**c**, dotted arrow)

**Fig. 3:**
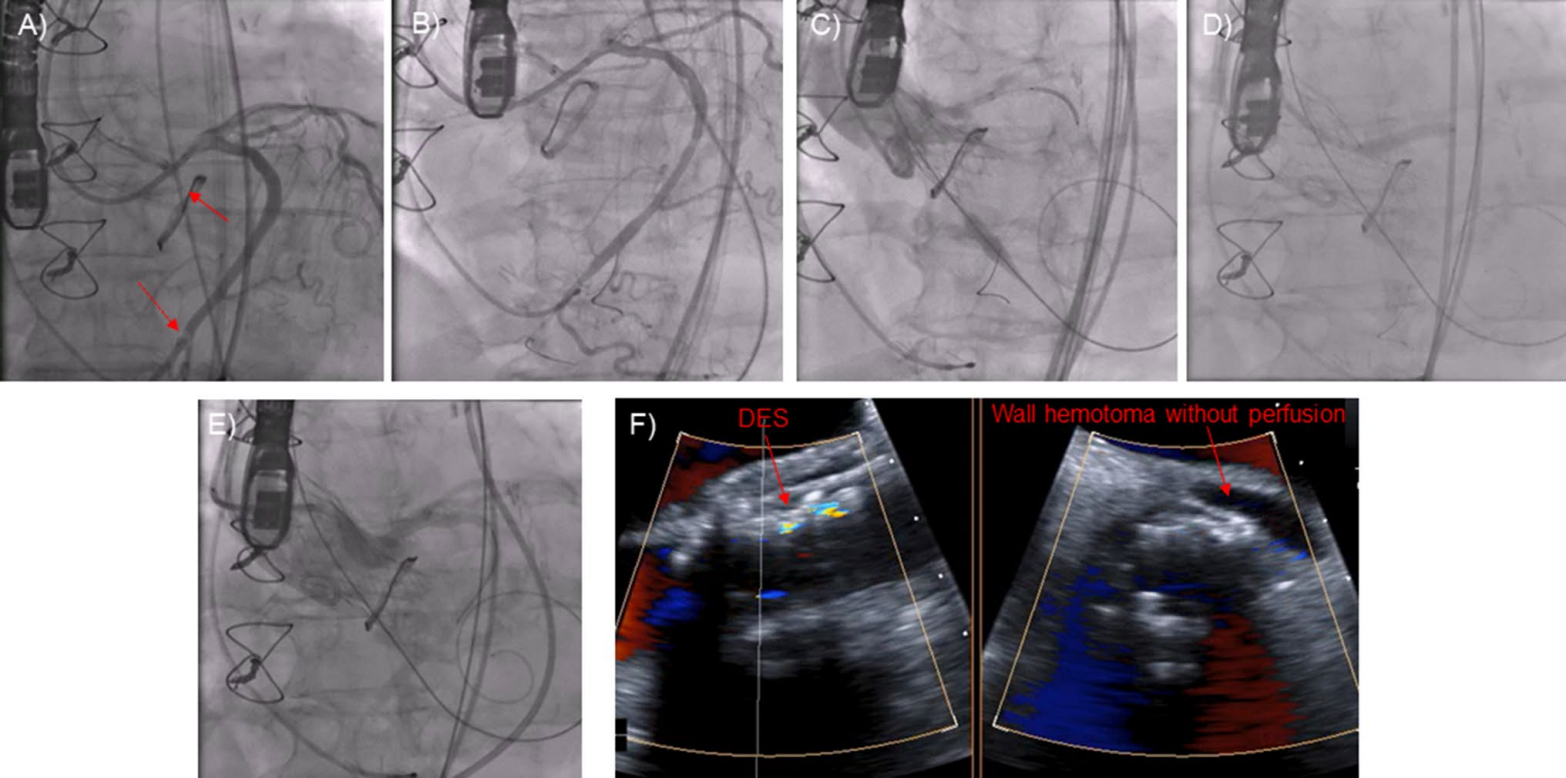
BASILICA procedure II. V-shape formation after successful traversal (**a**, solid arrow). Embolized material in the circumflex artery (**a**, dotted arrow) treated with a DES 3.5 × 16 mm (**b**). Prepositioning of another DES (4.0 × 24 mm), laceration of the left coronary cusp and implantation of an EvolutR 23 mm (**c**, **d**). Implantation of the stent with patent coronary flow and coverage of the laceration (**e**, **f**). *DES* drug-eluting stent

**Fig. 4:**
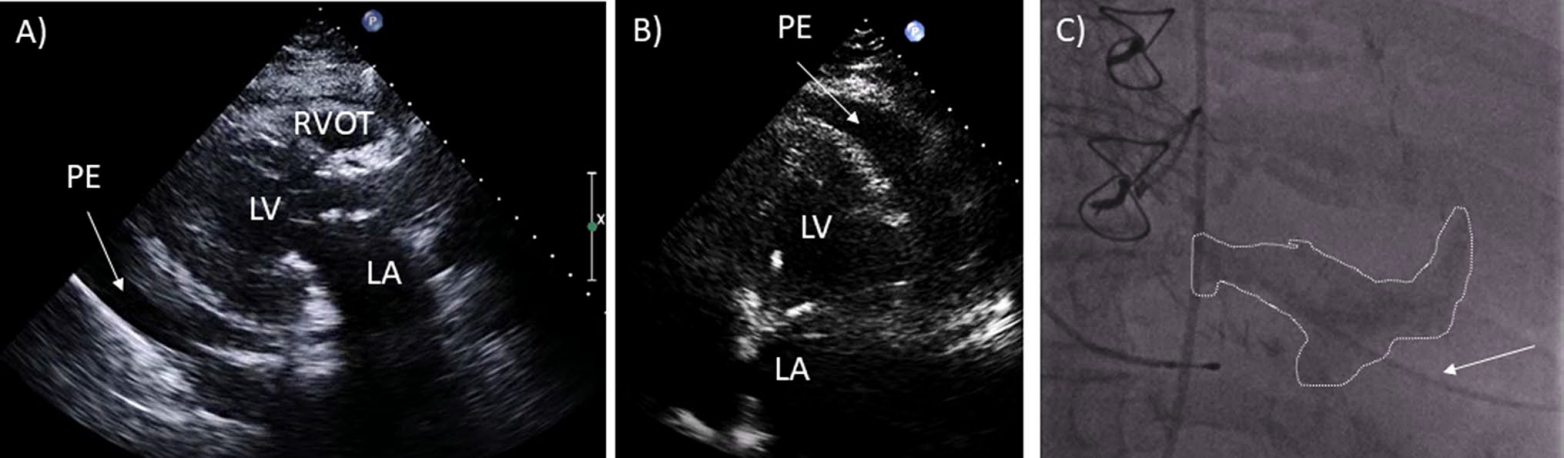
Pericardiocentesis. Posterior and lateral localized pericardial effusion (**a**, **b**). Echocardiography-guided apical puncture confirmed by contrast dye (dotted area) via a 5F dilatator (**c**, arrow). *LA* left atrium, *LV* left ventricle, *RVOT* right ventricular outflow tract, *PE* pericardial effusion

This case describes a potentially life-threatening complication of the BASILICA procedure that might be prevented by avoiding wire bending during the traversal attempt and prolonged energy application.

## Electronic supplementary material

Below is the link to the electronic supplementary material.

Supplementary file1 Video 1: Bending of the electrified wire causing laceration of the left Sinus of Valsalva confirmed by angiography and echocardiography. (AVI 1558 kb)

Supplementary file2 Video 2: Embolized material at the bifurcation of left circumflex and obtuse marginal treated by DES implantation. (MPG 4482 kb)

Supplementary file3 Video 3: Laceration of the left coronary cusp, TAVI implantation followed by DES implantation leading to patent coronary flow and coverage of the laceration. (AVI 6133 kb)
