# Correction to: An unintentional complication during an intentional procedure—Sinus of Valsalva laceration during BASILICA

**DOI:** 10.1007/s00392-021-01949-1

**Published:** 2021-10-26

**Authors:** Norman Mangner, Mohamed Abdel-Wahab, Krunoslav Sveric, Utz Kappert, Julia Fischer, Stephan Haussig, Axel Linke

**Affiliations:** 1 Department of Internal Medicine and Cardiology, Herzzentrum Dresden, Technische Universität Dresden, Fetscherstr. 76, 01307 Dresden, Germany; 2 Department of Structural Heart Disease/Cardiology, Heart Center Leipzig at University of Leipzig, Leipzig, Germany; 3 Department of Cardiac Surgery, Herzzentrum Dresden, Technische Universität Dresden, Dresden, Germany; 4 Dresden Cardiovascular Research Institute and Core Laboratories GmbH, Dresden, Germany

## Correction to: Clinical Research in Cardiology (2021) 110:754–757 10.1007/s00392-020-01745-3

The article An unintentional complication during an intentional procedure—Sinus of Valsalva laceration during BASILICA, written by Norman Mangner, Mohamed Abdel‑Wahab, Krunoslav Sveric, Utz Kappert, Julia Fischer, Stephan Haussig, Axel Linke was originally published Online First without Open Access. After publication in volume 110, issue 5, page 754–757 the author decided to opt for Open Choice and to make the article an Open Access publication. Therefore, the copyright of the article has been changed to © The Author(s) 2020 and the article is forthwith distributed under the terms of the Creative Commons Attribution 4.0 International License, which permits use, sharing, adaptation, distribution and reproduction in any medium or format, as long as you give appropriate credit to the original author(s) and the source, provide a link to the Creative Commons licence, and indicate if changes were made.

The images or other third party material in this article are included in the article’s Creative Commons licence, unless indicated otherwise in a credit line to the material. If material is not included in the article’s Creative Commons licence and your intended use is not permitted by statutory regulation or exceeds the permitted use, you will need to obtain permission directly from the copyright holder.

To view a copy of this licence, visit http://creativecommons.org/licenses/by/4.0/.

